# Delayed

**Published:** 1875-06

**Authors:** 


*Delayed.*

This issue of the RECORD has been delayed for some time because the paper upon which it is printed failed to reach the publisher. Owing to the peculiar quality of paper made—it being manufactured expressly for this journal—it could not be duplicated anywhere short of the mills where it is made. The publisher desires uniformity in paper, and had to wait, therefore, for the supply-order to reach us. Our friends will see the fault is not due to the editors, as the type has been up from the time it should have appeared. The publisher will stir the paper men up and come to time, we trust, for the future.

